# The Study of the History of Medicine

**Published:** 1903-07-25

**Authors:** 


### The Hospital.

A Journal of

The Medical Sciences and Hospital Administration.

Vol. XXXIV.—No. 878. Edited by Sir Henry Burdett, K.C.B. [July 25, 1903.

### The Study of the History of Medicine.

The first systematic course of lectures on the history of medicine was fitly entrusted to Dr. Joseph Frank Payne, the Harveian Librarian at the Royal College of Physicians of London. He chose Anglo-Saxon medicine as the subject of the FitzPatrick lectures and began them with the confession that our own country is behind most civilised nations in the bulk and value of its contributions to the history of medicine. "Germany, France, and Italy," he said, "have produced on this subject works both profound and brilliant, which remain our standard authorities. The smaller countries, such as Holland and Switzerland have not been behindhand; while our cousins across the Atlantic, the American physicians, are now displaying characteristic energy and zeal in the study of the antiquities of medicine." In England we have not a single institution in which the history of medicine is taught or considered, nor is there any society devoted to its study. Indeed so slight is the interest taken in the subject by the profession at large that only last year an attempt to form such a society proved abortive because less than a dozen persons could be found to indicate their adherence when the project was mooted. Yet our heritage is indeed a fair one, and would well repay the most careful cultivation. Nearly all the metropolitan hospitals and many of the country infirmaries have allowed their stories to be published in the form of extracts from the minute books of their committees, yet no one has yet thought it worth his while to study these publications as a whole in order to write a history of the medical corporations, hospitals and infirmaries in the United Kingdom—a kingdom which is unique in the fact that these institutions are supported by the gifts of individuals rather than by subsidies from the State.

Whole epochs of English medicine require careful study and are practically virgin ground. Where, for instance, did the better class of physicians and surgeons obtain their education in the early part of the fifteenth century, an education which so far opened their minds as to enable the two branches of the profession to combine and to work together for a time at least as harmoniously as do the conjoined colleges at the present day? What brought about the great advance in surgery during the Elizabethan period? An advance which raised surgery at once from a handicraft to a profession. Was it the result of the improved education given to the entire country by the destruction of the monasteries and the consequent setting free of large sums of money for the foundation of the grammar schools, or was it merely the action of a few large-minded individuals? Why did surgery decay so utterly under the Stuarts that Woodall could write truthfully in 1639. "For this forty years last past no surgeon of my nation hath published any book of the true practice of surgery to benefit the younger sort, these my mean treatises only excepted." The early connection too, between the medical faculties of Oxford and Paris; of England and Scotland with the Universities of Padua at a later date will well repay thorough investigation. Roger Bacon was a child of the first union; William Harvey of the second. The great Oxford school of the seventeenth century has never yet received adequate historical treatment, though the names of Harvey, Scarborough, Mayow, Lower, and Willis, with his demonstrators Wren and Millington, show how long would be the chapter which should give a due appreciation of its rise and fall. By what curious process of decadence did it come about that within a generation of this golden epoch all England should be discussing whether Mary Tofts, a poor woman at Godalming, was or was not the mother of live rabbits; surely such a sudden transition from knowledge to the most dense ignorance must be capable of some explanation? But no one has yet troubled himself to write of the contrast, much less to discover the causes.

The history of medicine opens up wide fields of national thought and action, for it will have to describe the wave of philanthropy which passed over the United Kingdom in the eighteenth century leading to the establishment of many of the general hospitals in London and to the founding of the county hospitals and infirmaries within a period of about fifty years. The history of the Scotch schools also is rich in incident and glorious in its recollection. The bitter quarrels of the medical giants in Edinburgh will certainly not prove unattractive reading now that the passage of time enables us to see the good in each and teaches us that they were working to a common end. The great names of the Dublin School, too, need greater recognition lest their lustre should become tarnished by lapse of time and the details of their lives lost by the death of individuals who had known them and learnt the art at their feet. Whole fields of work are waiting to be tilled in connection with the rise of the medical schools, the methods of teaching and the subjects taught. The gruesome interest attaching to dissection has caused the history of anatomy in England to be known pretty thoroughly but we are almost wholly ignorant of the early methods of teaching clinical medicine and surgery, nor do we know the means by which the older surgeons obtained their knowledge of operative work which was sometimes extensive and intricate as in the case of John of Arderne, the great fourteenth-century operator.

These are suggestions of what might be done in a well-considered history of medicine in the three kingdoms. But where all wants doing it is of but little use to point out details. The man who is to write a comprehensive history of English medicine must be at the same time young, learned, a good linguist especially "well seen," as the old writers would have it, in mediæval French and monkish Latin, and he must be well endowed pecuniarily, for the work he undertakes is the work of a lifetime, and it will bring him no remuneration beyond the knowledge of work well done.

